# Editorial: Signaling mechanisms of synapse assembly

**DOI:** 10.3389/fnsyn.2023.1154806

**Published:** 2023-02-15

**Authors:** Zhihui Liu, Richard Sando, Bo Zhang, Xiaofei Yang

**Affiliations:** ^1^Department of Molecular and Cellular Physiology, Stanford University, Stanford, CA, United States; ^2^Department of Pharmacology, Vanderbilt University, Nashville, TN, United States; ^3^Shenzhen Bay Laboratory, Institute of Neurological and Psychiatric Disorders, Shenzhen, China; ^4^School of Chemical Biology and Biotechnology, Peking University Shenzhen Graduate School, Shenzhen, China; ^5^College of Biomedical Engineering, South-Central Minzu University, Wuhan, China

**Keywords:** signaling mechanism, synapse assembly, inhibitory synapses, calcium channel, sepsis-associated encephalopathy (SAE)

As the most fundamental communication unit in the brain network, synapses are one of the hot topics in the field. In the past decade, numerous efforts have been invested into understanding the signaling mechanisms of synapse assembly, resulting in fruitful literature illuminating our understanding of synapse biology. This Research Topic aims to collect a series of studies on synapse formation research, especially inhibitory synapses.

In a research article, Imam et al. found a TC10-driven signaling mechanism in regulating the spatial organization of presynaptic neurotransmitter release at inhibitory synapses. Collybistin recruits gephyrin at inhibitory GABAergic synapses and interacts with small GTPases Cdc42 and TC10. The authors applied newly developed collybistin FRET sensors and investigated the molecular basis of the regulation of collybistin. Surprisingly, TC10 binds to closed/inactive collybistin, causing a switch from an auto-inhibited close state to an open/active state. This study suggests a new substrate in inhibitory synapse regulation.

Moreover, Boxer and Aoto summarized the role of neurexins (Nrxns) and their ligands at inhibitory synapses in a review. Neurexins are evolutionarily conserved presynaptic cell adhesion molecules, and they are encoded by three genes, Nrxn1-3. Functional studies of Nrxns have focused mainly on glutamatergic synapses. However, the role of neurexins' molecular functions at GABAergic synapses has been elucidated in recent studies. Neurexins express in brain-region, cell-type, and synapse-specific manner, therefore, they also exhibit brain-region, cell-type, and synapse-specific dependent functions. Meanwhile, neurexin expression at inhibitory synapses is regulated by activity. Lastly, Boxer and Aoto summarized the binding partners of neurexins at inhibitory synapses, including neuroligins, cerebellins, dystroglycan, neurexophilins, calsyntenin-3, carbonic anhydrase-related proteins 10 and 11 (CA10/11), FAM19A1-4, GABAAR, and immunoglobulin superfamily member 21 (IgSF21).

In another mini-review, Zhang et al. further summarized the progress of the signaling mechanism of voltage-gated calcium channels (CaV) at presynaptic active zones. Voltage-gated calcium channels at the presynaptic active zone are heterogeneously organized and highly diversified. CaV regulates the synapse functions not only by diverse CaV channel types but also by the expression of channel numbers. However, the organization is not chaotic or random. The spatial relationship between CaV channel clusters and synaptic vesicles is topographic and logical and can be modulated by G-protein coupled receptors.

Lastly, after a series of summaries and research on synapse signaling mechanisms under normal non-pathological conditions, Tang et al. discussed the synapse and synapse formation alterations under pathological conditions—sepsis-associated encephalopathy (SAE). Pathophysiological changes of SAE, including neuroinflammation, glial activation, and blood-brain barrier (BBB) breakdown, affect synapse formation and functions intensively. SAE inhibits synaptic plasticity, disrupts neurotransmitter balance, and damages myelin. Under SAE, synapse formation is largely affected due to the alternations of synaptic adhesion molecules, including neurexins and their ligands, and receptor protein tyrosine phosphatases. Synaptic modulation proteins, such as SYP, PSD95, and thrombospondin-1 (TSP1), also play vital roles during the process.

In summary, the articles on our Research Topic provide a broad view and perspective of the signaling pathway of synapse assembly under normal and pathological conditions. Extensive progress has been achieved in this field recently. Therefore, it is of great importance to organize and summarize the discoveries and findings. Even though a huge amount of work has been done and many candidate genes and signaling pathways have been uncovered, this is only the tip of the iceberg. Numerous unanswered questions remain to be addressed in order to understand the signaling mechanisms during synapse formation fully.

## Author contributions

ZL drafted the editorial. RS, BZ, and XY edited and provided inputs. All authors contributed to the article and approved the submitted version.

